# Androgen Excess Induced Mitochondrial Abnormality in Ovarian Granulosa Cells in a Rat Model of Polycystic Ovary Syndrome

**DOI:** 10.3389/fendo.2022.789008

**Published:** 2022-03-17

**Authors:** Linyi Song, Jin Yu, Danying Zhang, Xi Li, Lu Chen, Zailong Cai, Chaoqin Yu

**Affiliations:** ^1^ Department of Gynecology of Traditional Chinese Medicine, Changhai Hospital, Chinese People’s Liberation Army (PLA) Naval Medical University, Shanghai, China; ^2^ Department of Traditional Chinese Medicine, HwaMei Hospital, University of Chinese Academy of Sciences, Ningbo, China; ^3^ International Peace Maternity and Child Health Hospital, School of Medicine, Shanghai Jiao Tong University, Shanghai, China; ^4^ Department of Gynecology of Traditional Chinese Medicine, Integrated Traditional Chinese and Western Medicine of Jiangsu Hospital, Chinese Academy of Medical Sciences, Nanjing, China; ^5^ Department of traditional Chinese and Western medicine, Zhejiang Association of Traditional Chinese Medicine, Hangzhou, China; ^6^ Department of Biochemistry and Molecular Biology, Chinese People’s Liberation Army (PLA) Naval Medical University, Shanghai, China

**Keywords:** polycystic ovary syndrome, androgen excess, granulosa cells, mitochondria, mechanism

## Abstract

**Background:**

Androgen excess could profoundly lead to follicular dysplasia or atresia, and finally result in polycystic ovary syndrome (PCOS); however, the exact mechanism remains to be fully elucidated.

**Methods:**

PCOS model rats were induced by dehydroepiandrosterone, and their fertility was assessed. The ovarian granulosa cells (GCs) from matured follicles of PCOS model rats were collected and identified by immunofluorescence. The mitochondrial ultrastructure was observed by transmission electron microscope and the mitochondrial function was determined by detecting the adenosine triphosphate (ATP) content and *mtDNA* copy number. Besides, the expressions of respiratory chain complexes and ATP synthases in relation to mitochondrial function were analyzed.

**Results:**

The PCOS model rats were successfully induced, and their reproductive outcomes were obviously adverse. The GCs layer of the ovarian was apparently cut down and the mitochondrial ultrastructure of ovarian GCs was distinctly destroyed. The ATP content and *mtDNA* copy number of ovarian GCs in PCOS model rats were greatly reduced, and the expressions of *NDUFB8* and *ATP5j* were significantly down-regulated without obvious deletion of *mtDNA* 4834-bp.

**Conclusions:**

Androgen excess could damage mitochondrial ultrastructure and function of GCs in rat ovary by down-regulating expression of *NDUFB8* and *ATP5j* in PCOS.

## Introduction

Polycystic ovary syndrome (PCOS) is highly heterogeneously characterized by hyperandrogenism, polycystic ovaries, oligo-ovulation or anovulation, and irregular or absent menstrual cycles (1–3). It is a prevalent hormonal and metabolic disorder of premenopausal women worldwide, which accounts for 75% of anovulatory infertilities (4). The latest studies suggest that disordered follicular development is the core of the abnormalities that occur in the ovary in PCOS (5). Follicular development is a complex physiological process that is regulated by various substances and endocrine factors. During follicle growth, mural granulosa cells (GCs) play an active role in primary and secondary follicle development by secreting nutrients and hormones, and the cumulus GCs surrounding the oocytes play a crucial role in oocyte differentiation and regulation through the proliferation and production of energy source (6, 7). Nevertheless, a large number of sinus follicles are present in the bilateral ovaries of PCOS patients, but no mature follicles can be formed periodically, which is closely related to the original and intrinsic follicular dysplasia of these patients.

Androgens, one of the most important steroid hormones mainly produced by the adrenal glands and ovaries, play well-defined roles in female reproductive functions. Just like a double-edged sword as reported (8), besides the positive effects of androgen on follicular development, abnormal androgen levels, especially as androgen excess, profoundly promote follicular dysplasia or atresia. However, the underlying pathophysiological mechanisms remain largely unknown.

The mitochondria, which are referred to as the powerhouses and central to energy metabolism in cells (9), play a fundamental role in signal transduction for cell proliferation and apoptosis (10). They translate nutrients into available energy and release reactive oxygen species as by-products (11). Mitochondrial function is a key factor controlling female reproductive processes (12). Researchers have found that all steroidogenic pathways begin in the mitochondria (13, 14), and mitochondria participate in modulating human GCs steroidogenesis (15, 16). In this study, we attempted to investigate the effects and possible mechanisms of excessive androgen on mitochondria of follicular GCs by evaluating the PCOS model rats.

## Materials and Methods

### PCOS Model Rats’ Preparation and Reproductive Capacity Assessment

Wistar rats (SPF grade, female, age 3 weeks, body weight 45–55 g, *n* = 150) were provided by Beijing Viton Lever Laboratory Animal Co., Ltd. (license: SCXK [JING] 2016-0006). The rats were reared at 25°C, humidity of 45%–55% and light duration of 12 h. All rats were allowed free access to water and acclimatized for 2 days. This study was performed in PLA Naval Military Medical University Animal Center, and all animal procedures described here were reviewed and approved by the ethics committee of PLA Naval Military Medical University (permit number L2015035). According to the previous description (17, 18,) 100 rats were induced by subcutaneous injection of dehydroepiandrosterone (DHEA) (0.6 mg/100 g/day + 0.2 mL of sesame oil) for 20 consecutive days and verified by histological screening of vaginal exfoliated cells and hormonal profiles of the orbital venous blood. Meanwhile, another 50 rats were subcutaneously injected with sesame oil (0.2 mL/day) as control. 6 control group rats (with normal sexual cycles and hormones) and 6 PCOS model group rats (with abnormal sexual cycles and hormones) were randomly selected, and the ovarian morphology was observed by hematoxylin and eosin (HE) staining. In addition, 12 control group rats and 18 PCOS model group rats were randomly selected and caged with fertile males of the same strain (SPF grade, age 7 weeks, body weight 100–120 g, with previous reproductive history and adaptive feeding for 2 days as mentioned above) at a 1:1 ratio. Copulation was verified by the presence of a vaginal plug 24 h later, and this was considered gestational day (GD) 0.5. The non- pregnant rats were caged with male rats again on the second day, and the process was repeated three times in total, then the pregnancy cases of each group were counted, and the pregnancy rates were calculated. Moreover, half of the pregnant rats of both control group and PCOS model group were randomly selected and sacrificed between 08: 00 and 09: 00 hours on GD 18.5 (GD 18 is the middle and late stage of pregnancy), and the embryo outcomes including the total number of embryos, the average number of embryos, the average mass of embryos, and the number of absorbed embryos were assessed. The remaining pregnant rats in each group were reared until delivery, and then the litter size, the live birth and the abnormal situation of the pups were counted.

### Rat Ovarian GCs Collection and Identification

Both PCOS model group rats (DHEA-induced) and control group rats were injected subcutaneously with pregnant mare serum gonadotropin at a dose of 50 IU/rat. 48 hours later, the rats were sacrificed, and the ovaries were harvested. The mature follicles on the surface of the ovaries were pierced with a syringe under a dissecting microscope so that the cumulus oocyte complexes were released into the culture medium. Then, the medium was centrifuged (2000 g for 10 min at 4°C) and the GCs at the bottom of the Eppendorf tube were collected and purified by Ficoll density gradient centrifugation combined with the attachment culture method (6). The GCs were suspended at 37°C in a humidified incubator containing 5% CO_2_ in DMEM/F-12 supplemented with 15% FBS, 100 IU/mL of penicillin and 0.1 mg/mL of streptomycin. As is well known, the specific expression of follicle-stimulating hormone receptor (FSHR) is the appraisal standard, for the GCs are the only cells that express FSHR in ovarian tissue (19). In this study, Immunofluorescence was used to determine the presence and location of FSHR. Accordance with the previous study (20), after been seeded on coverslips in 24-well petri dishes 48 hour, the GCs were fixed with 4% paraformaldehyde for 20 min at room temperature and permeabilized with the use of 0.3% Triton X-100 for 40 minutes before incubating with 10% bovine serum albumin and FSHR antibody (1:200; Beyotime). Fluorescein isothiocyanate–conjugated IgG was used as the secondary antibody (1:100; ZSGB-BIO). The cells were then counterstained with DAPI and visualized under a fluorescence microscope (BX53; Olympus). The antibodies used here are provided in **Supplementary Table 1**.

### Rat Ovarian GCs Morphology and Ultrastructure Observation

Rat ovarian GCs suspensions of 6 PCOS model group rats and 6 controls were inoculated into 24-well petri dishes with coverslips, and the growth status was observed under an inverted phase contrast microscope at 48 hour after the GCs adherent to the coverslips. The coverslips with well-grown GCs were removed by ophthalmic tweezers made for HE staining. Six fields of each coverslip were captured under a microscope. Two investigators, blinded to the coverslip origin, independently analyzed the coverslips using the available photographs and calculated the results. In addition, the ultrastructure of rat ovarian GCs were observed using a transmission electron microscope (TEM). Following the references (21–23), the adherent ovarian GCs of both group rats (6 PCOS model ones and 6 controls) were digested by 0.25% trypsin (without EDTA) and centrifuged (2000 g for 5 min at 4°C). The cell pellets were fixed with glutaraldehyde (2.5% in 0.1 mol/L cacodylate buffer, pH 7.2) (Fuchen, China) for 2 h at 4°C and post-fixed in 1% osmium tetroxide (Ted Pella, USA) for 1 h before being dehydrated in acetone and embedded in SPI-Pon-812 (SPI Supplies, USA). After slicing by using an ultramicrotome (Leica EM UC7), the 0.1 mm thin sections were stained with uranyl acetate and lead citrate and then observed under a TEM (Hitachi HT7700, 120kV).

### Rat Ovarian GCs ATP Content Measurement and *mtDNA* Copy Number Quantification

The Adenosine triphosphate (ATP) content of ovarian GCs in 6 PCOS model group rats and 6 controls were measured through utilization of recombinant firefly luciferase and its substrate D-luciferin following the ATP Assay Kit (Beyotime, China) instruction. Specifically, fresh ovarian GCs of each rat either control or PCOS model group were washed in culture medium and cultured in 96-well petri dishes; 24 hours later the adherent GCs were collected and washed with precooled phosphate buffer and lysed in ATP lysis buffer, and centrifuged at 12,000 g for 10 minutes at 4°C. Then, the supernatant was mixed with the testing buffer and quantified, and the ATP content was measured using a GloMax Luminescence detector (Berthold, Germany) according to the manufacturer’s instructions (23), Luminescence was monitored on an illuminometer with a maximum emission of 560 nm, and the ATP concentration was calculated using the ATP standard curve. Besides, the ovarian GCs of another 6 PCOS model group rats and 6 controls were prepared, the total DNA of GCs in each rat was extracted by incubating GCs with DNA extraction buffer (Omega, USA) at 55°C followed by heating at 98°C for 10 min, according to the previous report (24), the mitochondrial DNA (mtDNA) copy number was quantified by Bicinchoninic Acid Assay (BCA) and determined by Real-time PCR using a SYBR green assay on a 7500 Real-Time PCR system (Applied Biosystems, USA). The single copy gene mt-ND1 in mitochondrial *mtDNA* and the housekeeper gene β-globin in nuclear *nDNA* were amplified by Real-time PCR. β -Globin sequence was considered as the reference gene for its characteristic of highly conserved and expressed statically, and the changes of *mtDNA* copy number in both samples were compared. Intriguingly, the *mtDNA* 4834- bp deletion in rats, similar in size and location to the *mtDNA* 4977- bp deletion in humans, occurs frequently in various tissues and increases in an age-related manner (25). Therefore, the quantity of *mtDNA* 4834-bp deletion was determined by coamplifying the *mtDNA* displacement-loop (D-loop) and *mtDNA* 4834-bp deletion in a Real-time PCR assay in our study. Following the references (26), PCR amplification was carried out in a 20-*μ*L reaction volume consisting of TaqMan Universal Master mix (4 *μ*L), 200 nmol/L each *mtDNA* 4834-bp deletion primer, 50 nmol/L D-loop primer, and 100 nmol/L *mtDNA* 4834-bp deletion and D-loop probe primer. The cycling condition included an initial phase of 2 min at 50°C, 10 min at 95°C, then 40 cycles of 15 s at 95°C and 0.5 min at 72°C. The relative *mtDNA* copy number was calculated using the 2-ΔΔCt method. The primer sequences were designed and synthetized by Invitrogen (Invitrogen Biological Technology Co., Ltd, Shanghai, China) and listed in **Supplementary Table 2**.

### Rat Ovarian GCs Mitochondrial Functional Gene Analysis

To explore the possible mechanisms of excessive androgen (DHEA) on mitochondria of follicular GCs, 6 PCOS model group rats and 6 control group rats were randomly collected, and their ovarian GCs were harvested. Total *mRNA* was extracted from GCs in each rats using TRIzol reagent, and reverse transcription was performed using the Reverse Transcription cDNA Kit (Takara, Japan) following the manufacturer’s instructions. The resulting cDNA was diluted 10-fold in sterile water, and aliquots were subjected to Real-Time PCR. Finally, the mitochondrial functional related genes including ATP synthase subunits (*ATP5h*, *ATP5j* and *ATP5a1*), Dynamin-related GTPase (*Opa1*), Succinate dehydrogenase [ubiquinone] iron-sulfur subunit (*SDHB*), NADH dehydrogenase [ubiquinone] 1 beta subcomplex subunit 8 (*NDUFB8*), Cytochrome b-c1 complex subunit 2 (*UQCRC2*), Cytochrome c oxidase subunit 2 (*COXII*), NADH dehydrogenase [ubiquinone] flavoprotein 2 (*NDUFV2*) and Mitofusin-1 (*Mfn1*) mRNA levels were analyzed by quantitative PCR instrument (ROTORGENE6000, Carbett). The relative expression of each target gene compared with GAPDH was calculated using the 2-ΔΔCt method. Moreover, the proteins encoded by mRNAs that are significantly down-regulated in the PCOS model rats were further confirmed by western blot analysis. The adherent GCs was collected when their growth reached to 80% and lysed in cell lysis buffer (Thermo Fisher Scientific, USA) containing a protease inhibitor cocktail (Roche, Switzerland) for 30 min at 4°C. The bicinchoninic acid (BCA) assay (Thermo Fisher Scientific, USA) was used to determine protein concentrations. 40μg protein from each sample was separated by 12% sodium dodecyl sulphatepolyacrylamide gel electrophoresis (SDS-PAGE) (Invitrogen, USA) and transferred onto polyvinylidene fluoride membranes (Roche, Switzerland) for 1.5 h. The membranes were incubated overnight at 4°C with anti- oxidative phosphorylation (OXPHOS) and anti-GAPDH (Proteintech, USA) antibodies after 1 h in blocking buffer containing 5% non-fat dry milk and 0.1% Tween 20 in Tris-buffered saline (TBST). After washed 3 times with TBS-T, the membranes were incubated with secondary antibodies (Proteintech, USA) at room temperature. The protein bands were detected using an enhanced electrochemiluminescence (ECL) Detection System (Thermo Fisher Scientific, USA) and analyzed with the Fusion Solo system (Vilber Lourmat, France). The primer sequences were listed in **Supplementary Table 3** and the antibodies used in western blot were listed in **Supplementary Table 1**.

### Statistical Analysis

Continuous variables are presented as mean ± standard deviation (SD) and categorical variables are described as the numbers (percentages). The *student’s t-test* was applied for comparisons of the mean between the two groups if the variables are normally distributed; otherwise, *rank sum test* was performed. *F test* was utilized for the Equality of Variance test. *Fisher’s exact test* was applied when the number of categorical variables was less than 5 in each group and Satterthwaite was applied when the continuous variables were skewed. Analyses were performed using GraphPad Prism software version 8 (Graph-Pad software, San Diego, CA), and the statistical analysis was conducted using SPSS software, version 22.0 (SPSS, Inc., Chicago, IL, USA). P-values < 0.05 were considered statistically significant and all tests were two-tailed.

## Results

### The Reproductive Capacity of PCOS Model Rats Induced by DHEA Was Decreased

**Figure 1:**
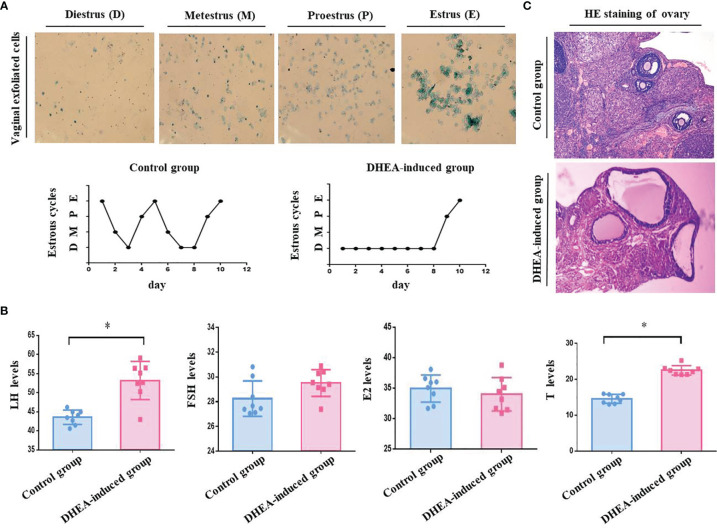
**(A)** Vaginal exfoliated cells of rats were observed under a microscope. As shown in the pictures, large numbers of leukocytes and a small amount of mucus are observed in Diestrus (D). In Metestrus (M), several types of cells are observed, including nucleated epithelial cells, keratin epithelial cells and leukocytes. In Proestrus (P), most cells are nucleated epithelial cells. In Estrus (E), large numbers of keratin epithelial cells are observed. The estrous cycle of rats in the control group last 4- 5 days, whilst that of rats in PCOS model group (DHEA induced) was disordered or even remained in the estrous interphase. **(B)** Serum sex hormones in the orbital vein of rats detected by ELISA. The sex hormones including LH, FSH, T and E2 were detected after DHEA induction. According to the result of ELISA, the serum levels of LH (53.19 ± 7.6 mIU/mL) and T (22.53 ± 4.7 nmol/L) in the PCOS model group rats was significantly increased compared with those in the control group (LH, 43.55 ± 6.7 mIU/mL; T, 14.60 ± 4.4 nmol/L), there were no obviously difference in the serum level of E2 (PCOS, 34.01 ± 9.7 pmol/L *vs* Control, 34.96 ± 7.9 pmol/L) and FSH (PCOS, 29.52 ± 3.8 mIU/mL *vs* Control, 28.26 ± 5.1 mIU/mL) between two groups. **(C)** Pathological morphology of rat ovarian tissues stained with HE. As shown in photos, no structural abnormalities were present in the control group rats: the ovarian tissue was pink, the follicles and corpora lutea were in varying stages of development, and number of GC layers was 6–8. Differences in PCOS model group rats were significant: the color of ovarian tissue was generally lighter, the number of cystic follicles (large fluid-filled cysts) was increased, and the GC layers were abnormal (only 2–4 or even less), and the number of corpora lutea sharply decreased. *P < 0.05.

Our studies showed that PCOS model group rats were sexual cycle disorder and endocrine abnormalities according to the classification of vaginal exfoliated cells and the detection of serum sex hormones. The success rate of PCOS rat model induced by DHEA was 75% (**Figures 1A, B** and **Table 1**). PCOS model group rats exhibited physical changes, for example, their hair was less glossy, coarser and harder in texture, and they behaved irascibly and even fought compared with control rats. Moreover, the ovarian pathological morphology of PCOS model group rats was greatly altered. For instance, the number of cystic follicles (large fluid-filled cysts) increased, and GCs layers were decreased sharply (**Figure 1C**). According to our calculation, the pregnancy rate and embryo outcome on GD 18.5 of PCOS model group rats, including the total embryos number, average number of embryos, average weight of embryos, litter size and the average litter size were significantly lower than those of the control group rats (*P* < 0.05). Besides, absorbed embryos generally refer to a condition in which the embryos are absorbed due to obstacles in its development, that is, the pregnant rats absorb some embryos to reduce their numbers and preserve the development of the rest when the reproductive conditions worsen for the development of embryos. In our study, the number of absorbed embryos in PCOS model group rats was obviously higher than that in control group rats (*P* < 0.05), which was consistent with the results reported in the literature (27) (**Tables 2**–**4**). To sum up, the fertility of PCOS model rats induced by DHEA was significantly decreased.

**Table 1:** The success rate of PCOS rat model induced by DHEA.

Group	Method (Subcutaneous injection)	Total number	Sexual cycle disorder number	Success rate
The control group	sesame oil (0.2mL/day)	50	0	0.00%
The model group	DHEA (0.6mg/100g/day dissolved in 0.2mL of sesame oil)	100	75	75.00%*

compared with the control group, *P < 0.05.

**Table 2:** The pregnancy rate of the rats.

Group	Number	Cage (the first time)		Cage (the second time)		Cage (the third time)		Total Pregnancy rate
		Pregnancy number	Pregnancy rate	Pregnancy number	Pregnancy rate	Pregnancy number	Pregnancy rate	
**The control group**	12	8	66.67%	3	75.00%	1	100.00%	100.00%
**PCOS model group**	18	2	11.11%*	5	31.25%*	4	36.36%*	61.11%*

compared with the control group, *P < 0.05.

**Table 3:** The embryo outcomes of rats on GD 18.5.

Group	Number	Total embryo number	Average number of embryos	Average mass of embryos	Number of absorbed embryos
**The control group**	6	212 (m_0.5_ 16.5)	16.17 ± 2.32	5.35 ± 0.38	1
**PCOS model group**	5	98 (m_0.5_ 8)	7.80 ± 3.77*	4.94 ± 0.47*	6

6 pregnant rats were randomly selected from the control group and 5 pregnant rats were randomly selected from the model group, these 11 pregnant rats were sacrificed on GD 18.5, and the condition of the embryo analyzed. Compared with the control group, *P < 0.05.

**Table 4:** The live births of progeny in rats.

Group	Number	Litter situation		Live birth situation		Abnormal situation	
		Litter size	Average litter size	live birth number	live birth rate	Malformation number	Malformation Rate
**The control group**	6	114 (m_0.5_ 19)	19.00 ± 3.41	114 (m_0.5_ 19)	100.00%	0	0.00%
**PCOS model group**	6	53 (m_0.5_ 9)	8.83 ± 4.75*	53 (m_0.5_ 9)	100.00%	0	0.00%

the rest 6 pregnant rats in the control group and the rest 6 pregnant rats in the PCOS model group were raise until they give birth; the condition of the pups is counted. Compared with the control group, *P < 0.05.

### Rat Ovarian GCs FSHR Immunofluorescence Identification and Morphological Observation

**Figure 2:**
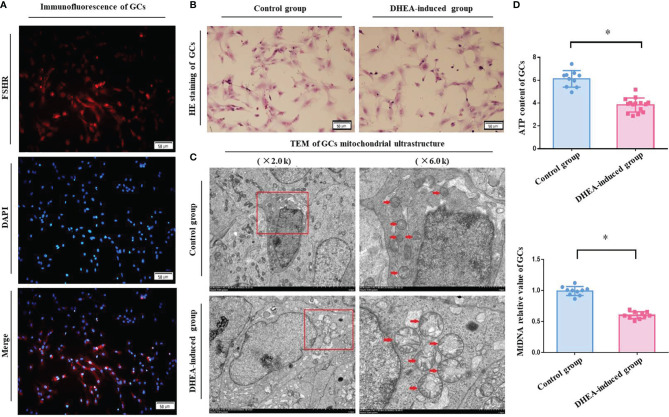
**(A)** The specific expression of FSHR in rat ovarian GCs was identified by immunofluorescence. The fluorescent microscopy (Leica) results showed a specific red fluorescence in the cytoplasm of positive cells, blue fluorescence in the nucleus of negative control cells and no red fluorescence in the cytoplasm. Merge represents the fusion of two colored images. **(B)** Morphological observation of rat ovarian GCs stained with HE. HE staining coverslips showed that the rats ovarian GCs were intact with pink cytoplasm and red-purple round nuclei, and there were no obvious difference between two groups under light microscopy. **(C)** The mitochondrial ultrastructure of rat ovarian GCs were analyzed by TEM. As shown by the red arrow in the photos, GCs had a normal nucleus with a clear nucleoli and double nuclear membranes, and the mitochondria had a clear mitochondrial crista around the nucleus in control group. However, the ovarian GCs presented mitochondrial aggregated distribution, crista dissolution and fracture and mitochondria with vacuoles in PCOS model group rats. **(D)** The ATP content and *mtDNA* copy number of rat ovarian GCs were detected. According to the analysis, the ATP content (4.51 ± 0.49 μm) and the *mtDNA* copy number (0.58 ± 0.12) of the ovarian GCs in PCOS model group rats were significantly decreased compared with those in control group rats (ATP, 6.53 ± 0.41 μm; *mtDNA*, 1.00 ± 0.19). *P < 0.05.

For further evaluation, ovarian GCs of rats were obtained, and FSHR immunofluorescence showed that specific red fluorescence was observed in the cytoplasm of positive cells, blue fluorescence in the nucleus of negative control cells and no red fluorescence in the cytoplasm. The proportion of cells with positive FSHR expression was more than 95%, which met the requirements of subsequent experiments (**Figure 2A**). After incubation for 48 h, ovarian GCs were adhered to the culture dish and forming a single layer of star-shaped or fusiform cells. HE staining sections showed that the cells were intact with pink cytoplasm and red-purple round nuclei, and there were no obviously visual differences between the control and PCOS model group rats (**Figure 2B**).

### The Mitochondrial Ultrastructure of Ovarian GCs in PCOS Model Rats Was Abnormal

The TEM images of the ultrastructure of rat ovarian GCs are shown in **Figure 2C**. In control group rats, all the ovarian GCs had a normal nucleus with a clear nucleoli and double nuclear membranes, and the mitochondria had a clear mitochondrial crista around the nucleus. However, the ovarian GCs of 4 PCOS model rats presented mitochondrial aggregated distribution, crista dissolution and fracture and mitochondrial vacuoles. It was suggested that the mitochondrial structure of GCs in ovary of PCOS model rats induced by DHEA was obviously abnormal, and the incidence of abnormality was 66.66% (**Table 5**).

**Table 5:** The incidence of abnormal mitochondrial structure .

Group	Total rat Number	number of rat with normal mitochondrial structure	number of rat with abnormal mitochondrial structure	abnormal structure rate
**The control group**	6	6	0	0.00%
**PCOS model group**	6	2	4	66.66%*

6 rats were respectively and randomly selected from the control and PCOS model group rats. The ovarian GCs of both groups’ rats were harvested, and their mitochondrial ultrastructure was observed by TEM. Compared with the control group, *P <0.05.

### The ATP Content and *mtDNA* Copy Number of Ovarian GCs in PCOS Model Rats Were Reduced Without *mtDNA* 4834- bp Deletion

**Figure 3:**
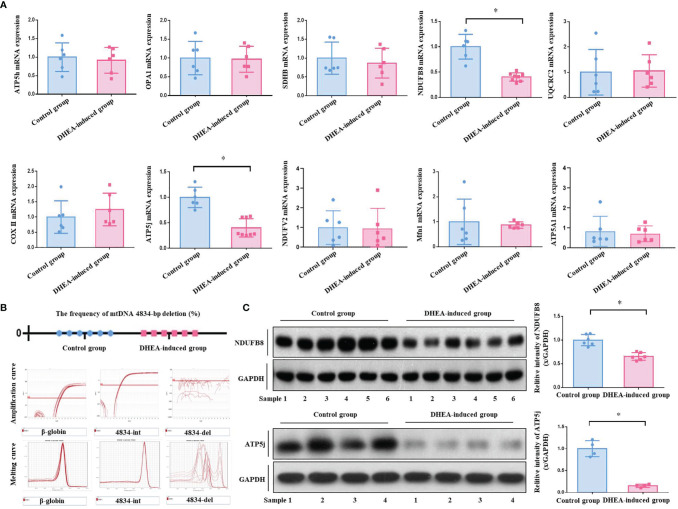
**(A)** The *mRNA* expression of mitochondrial functional genes in rat ovarian GCs was detected. A total of 10 mitochondrial functional related genes were analyzed by Real-time PCR. The results showed that the *mRNA* expressions of gene *NDUFB8* (0.40 ± 0.08) and *ATP5j* (0.40 ± 0.17) in PCOS model rats were significantly decreased compared with those in control group ones (*NDUFB8*, 1.00 ± 0.22; *ATP5j*, 1.00 ± 0.18). However, other genes including *SDHB, ATP5h, ATP5a1, Opa1, UQCRC2, COXII, NDUFV2 and Mfn1* were not obviously different between two groups. **(B)** Frequency of *mtDNA* 4834-bp deleted mitochondria detected. According to Real-time PCR analysis, there was no one with *mtDNA* 4834-bp deletion in both control and PCOS model group rats. The frequency of *mtDNA* 4834-bp deletion in both two groups was 0.00%. According to the amplification curve and melting curve, the int primer pair had specific amplification in the PCOS model group, while the del primer pair had no specific amplification, verified the results from both positive and negative perspectives. **(C)** The protein expressions of mitochondrial functional genes *NDUFB8* and *ATP5j* were detected. According to Western Blot analysis, the protein expression of mitochondrial functional genes *NDUFB8* and *ATP5j* in the ovarian GCs of the PCOS model group rats were obviously decreased compared with those in the control group. The results were consistent with those of Real-time PCR analysis. *P < 0.05.

According to the analyses of ATP bioluminescence detection kit and Real-time PCR, the ATP content (4.51 ± 0.49 μm) and *mtDNA* copy number (0.58 ± 0.12) of ovarian GCs in PCOS model group were significantly reduced compared with the control group (ATP, 6.53 ± 0.41 μm, *P <*0.0001; *mtDNA*, 1.00 ± 0.19, *P* = 0.0022) (**Figure 2D**). Real-time PCR analysis results showed that there was no one with *mtDNA* 4834-bp deletion in both control and PCOS model rats. The frequency of *mtDNA* 4834-bp deletion in both groups was 0.00%. According to the amplification curve and melting curve, the int primer pair had specific amplification in PCOS group, while the del primer pair had no specific amplification, verified the results from both positive and negative perspectives. (**Figure 3B**). It indicated that there was mitochondrial dysfunction in ovarian GCs of PCOS model rats induced by DHEA, with no related to the absence of mitochondrial fragments, in other words, it is not associated with the natural aging.

### The Expression of Mitochondrial Functional Genes NDUFB8 and ATP5j in PCOS Model Rat Ovarian GCs Were Significantly Reduced

Real-time PCR results showed that the *mRNA* expressions of *NDUFB8* and *ATP5j* genes in the PCOS model group rats were significantly decreased compared with the control group rats (*P* < 0.05), whilst the *mRNA* expressions of other genes including *SDHB*, *ATP5h*, *ATP5a1*, *Opa1*, *UQCRC2*, *COXII*, *NDUFV2* and *Mfn1* were not obviously different between two groups (*P* > 0.05) (**Figure 3A**). For further verification, the protein expression of *NDUFB8* and *ATP5j* was analyzed by Western blot. As shown in **Figure 3C**, the protein expressions of *NDUFB8* and *ATP5j* were obviously down-regulated in PCOS model group rats, the results of which were consistent with the Real-time PCR analysis.

## Discussion

Oligo-ovulation or anovulation is one of the most important clinical features of PCOS, which is the main cause of irregular menstruation and infertility. Ovulation induction by clomiphene or letrozole is the first-line treatment for PCOS infertility; however, the pregnancy rate is only 40%, and approximately 50% of PCOS infertility patients have to resort to assisted reproductive technology to solve fertility problems (9). Nevertheless, numerous intractable problems and challenges still remain, including the low fertilization and cleavage rate, decreased oocyte quality, and increased immature follicle rate. The fundamental reason for this is that the original and intrinsic follicular dysplasia of PCOS patients. Follicular development is a complex physiological process, wherein the growth of GCs is an important symbol. GCs are monolayer or multilayer cuboidal epithelial cells attached to the luminal surface of the follicle and play an important role in follicular steroid hormone secretion, energy metabolism (28) and conduct bidirectional information and material exchange with oocytes through gap junction in follicles (29, 30). Any problems of GCs in metabolism, growth differentiation and apoptosis will directly or indirectly affect the quality of follicular (31). Androgens, although traditionally thought to be male sex steroids, are involved in regulating dynamic changes in ovarian steroidogenesis that are critical for normal cycling in females (32). Current scientific consensus is that a careful balance of androgen activity in the ovary is necessary for reproductive health in women. Both androgen insufficiency and androgen excess could cause ovarian dysfunction. Although lack of androgen activity in the ovary leads to ovarian insufficiency as reported (33), androgen excess is linked to follicular dysplasia and appears to be both a cause and a consequence of PCOS in a vicious cycle (32). However, the exact mechanism remains to be fully elucidated.

The mitochondria, the main organelle of energy production and aerobic respiration in cells, are involved in many aspects of function regulation and have been demonstrated as key factors controlling female reproductive processes (34). As is well known that mitochondria are critical sites for steroid hormone biosynthesis, the initiation and rate-limiting step in the biosynthesis of steroid hormones is the transfer of cholesterol to mitochondria, which is promoted by Steroidogenic acute regulatory protein (35). Therefore, functional mitochondria were demonstrated to facilitate GCs steroidogenesis and dysfunctional mitochondria of GCs may contribute to the global decline of steroidogenesis, oocyte maturation rate, and fertilization rate, and it can ultimately jeopardize fertility (12). Nucleus deoxyribonucleic acid and *mtDNA* encode mitochondrial respiratory chain-related proteins in mitochondrial organelles that produce cellular energy (ATP) through oxidative phosphorylation (OXPHOS). *MtDNA* gene encodes 2 ribosomal RNAs (12S rRNA and 16s rRNA), 22 transporter RNAs, 13 oxidative phosphorylated protein subunits and an independent D-loop region. These proteins play an important role in the respiratory chain complex of the OXPHOS system, facilitating the synthesis of ATP by ATP synthase (F0F1-ATPase) and providing energy support for the normal functioning of cells (36). The *MtDNA* copy number is essential for maintaining mitochondrial function and cell growth (37) and is an important means to reflect mitochondrial metabolic function accurately in the study of disease phenotypes (38). Ogino et al. found that the *mtDNA* copy number of follicular GCs could predict embryo quality during *in vitro* fertilization (39). In our preliminary clinical studies, we found that the ATP content and *mtDNA* copy number of ovarian GCs in PCOS patients, especially those with hyperandrogenism, were obviously decreased compared with those non-PCOS patients. However, the effects and possible mechanisms of androgen excess on mitochondria of ovarian GCs have not been reported so far. In this research, PCOS model rats were induced by subcutaneous injection of DHEA and their fertility was obviously reduced according to the assessment. Besides, the mitochondrial structure and function of ovarian GCs in PCOS model rats were investigated. Mitochondrial structure disorder is important cause of disease (40). As the TEM showed that the mitochondrial structure of rat ovarian GCs was seriously damaged after DHEA intervened. Notably, the ATP content and *mtDNA* copy number of ovarian GCs in PCOS model rats were obviously reduced.

The deletion of 4977-bp and 4834-bp DNA fragments of the mitochondria is common in humans and rats, respectively, and the deletion rate increases gradually with age (25). The 4977-bp DNA fragment was found to be deleted in GCs of elderly women, which was associated with the reduction of ATP (41). However, no relevant studies have been reported on the deletion of the *mtDNA* fragment in ovarian GCs of PCOS. In our study, no obvious deletion of the 4834-bp fragment was found in ovarian GCs of PCOS model rats induced by DHEA. Thus, we speculated that the decrease of ATP in ovarian GCs of PCOS model rats is not associated with the deletion of *mtDNA* fragments, which means it doesn’t matter with the age. For further exploration, the molecular regulatory mechanisms of mitochondrial function were studied in our research. Firstly, mitochondrial fusion function is significant to the quality and longevity of mitochondria, and *Mfn1* and *OPA1* are important regulators of mitochondrial fusion function. *OPA1* regulates mitochondrial endometrial fusion and affects oxidative phosphorylation (42); *Mfn1* regulates mitochondrial outer membrane fusion and affects mitochondrial distribution (43). However, neither *Mfn1* nor *OPA1* showed obvious imbalance in the expression and interaction according to our study results. Secondly, the activity of the OXPHOS system, which consists of four electron transport chain complexes (complex I to IV), F0F1-ATPase (complex V) in the mitochondrial inner membrane and enzymes regulating the tricarboxylic acid cycle and substrate metabolism, is important for ATP synthesis. In our study, the expression of *NDUFB8*, an adjunctive subunit of mitochondrial membrane respiratory chain NADH dehydrogenase (complex I) (44), was significantly decreased in ovarian GCs of PCOS rats induced by DHEA. Nevertheless, other OXPHOS complex subunits including *COX2*, *SDHB*, *UQCRC2* and *NDUFV2* were not obviously changed. *ATP5j*, *ATP5a* and *ATP5h* are key enzymes to enhance OXPHOS action and promote ATP synthesis in the mitochondrial inner membrane. Amongst them, *ATP5j*, which encodes the F6 subunit of the F0 complex, is one of the constituent subunits of F0F1-ATPase and plays a key role in the connection of F0 and F1 (45). In the present study, the expression of *ATP5j* was significantly down-regulated in ovarian GCs of PCOS rats induced by DHEA.

There are some limitations in the current research. First of all, a significant decrease of ovaries GCs was observed in the PCOS model rats induced by DHEA. As shown in histology, the number of cystic follicles (large fluid-filled cysts) was obviously increased, and the GC layers were visibly abnormal (only 2–4 or even less). To obtain a sufficient number of follicular GCs for further study, we induced superovulation with PMSG, a commonly used gonadotropin. There is no doubt that PMSG will increase a certain number of natural follicles, as well as a certain number of natural GCs. In practice, however, it is difficult to distinguish the natural GCs caused by PMSG from the unhealthy GCs caused by DHEA. To be more scientific and rigorous, and avoid the potential of bias causes by PMSG, a control group was specially set up in our research, consistently, the control group rats were given the same dose of PMSG at the same time, and a series of subsequent studies on the mitochondrial structure and function of ovarian GCs were carried out on this basis. Nevertheless, the baseline of total energy of GCs was increased in both groups. What’s more, there has not been an extensive and comprehensive screening of mitochondrial functional genes. It is well known that there are many genes in the OXPHOS system, some of which are from mitochondrial DNA and others are genomic. Genes from complex I, II, III, IV and V and fusion genes were selected and explored in this study. Although all of these candidate genes are functionally accurate genes that have been extensively studied and widely reported in the literatures, more studies on TCA cycle, mitophagy and fission genes with ROS measurement are needed to get more sense for mitochondrial function.

## Conclusion

In summary, our research revealed that the mitochondria of ovarian GCs in PCOS rats showed a disordered structure and dysfunction, and the failure of mitochondrial function might be associated with the down-regulation of the expression of *NDUFB8* and *ATP5j* by excessive androgen.

## Data Availability Statement

The datasets presented in this study can be found in online repositories. The names of the repository/repositories and accession number(s) can be found in the article/**Supplementary Material**.

## Ethics Statement

The animal study was reviewed and approved by The Ethics Committee of PLA Naval Military Medical University (permit number L2015035). Written informed consent was obtained from the owners for the participation of their animals in this study.

## Author Contributions

LS and JY designed the study, conducted the entire experiment, collected the data, and drafted the original version of manuscript. DZ and XL contributed to statistical analyses. LC offered intellectual support for the framework of the study. CY and ZC are the guarantors of this work, supervised the data collection, and accept full responsibility for the conduct of the study. All authors contributed to the article and approved the submitted version.

## Funding

This work was supported by the National Natural Science Foundation of China (grant numbers 81973896, 81603646), China Postdoctoral Science Foundation (grant numbers 2020M681337) and Special Projects of Military Medical Research of Changhai Hospital (grant numbers 2018JS018).

## Conflict of Interest

The authors declare that the research was conducted in the absence of any commercial or financial relationships that could be construed as a potential conflict of interest.

## Publisher’s Note

All claims expressed in this article are solely those of the authors and do not necessarily represent those of their affiliated organizations, or those of the publisher, the editors and the reviewers. Any product that may be evaluated in this article, or claim that may be made by its manufacturer, is not guaranteed or endorsed by the publisher.
