# Supplementary material for: Androgen Excess Induced Mitochondrial Abnormality in Ovarian Granulosa Cells in a Rat Model of Polycystic Ovary Syndrome
Source: Front Endocrinol (Lausanne). 2022 Mar 17;13:789008. doi: 10.3389/fendo.2022.789008 (PMC8967935; doi:10.3389/fendo.2022.789008)
Supplement: Supplementary file 1 [file Table_1.docx]

Supplementary Material

# Supplementary tables

**Supplementary Table 1. Antibodies used in this research**.

| Antibody | Host species | Source | Dilution |
| --- | --- | --- | --- |
| FSHR | rabbit | Beyotime | 1:200 |
| Fluorescein isothiocyanate–conjugated IgG |  | ZSGB-BIO | 1:100 |
| NDUFB8 | mouse | Proteintech | 1:1000 |
| Anti-mouse IgG(HRP) |  | CST | 1:5000 |
| ATP5j | rabbit | Proteintech | 1:1000 |
| Anti-rabbit IgG(HRP) |  | CST | 1:5000 |

**Supplementary Table 2. The primer sequences of target genes**

**in mtDNA copy number quantification.**

| The name of the primer | primer sequences (5’→3’) |
| --- | --- |
| r-N-β-2-microglobin-F | CGGGGTGGTGATGAGAAGTT |
| r-N-β-2-microglobin-R | AAGGCTCCTTGTCCCTTGAC |
| MT-ND1-F | AGGCTTCGGGAACTGACTTG |
| MT-ND1-R | AGATAGAAGACACCCCGGCT |
| 4834-int-F | GGTTCTTACTTCAGGGCCATCA |
| 4834-int-R | GATTAGACCCGTTACCATCGAGAT |
| 4834-del-F | AAGGACGAACCTGAGCCCTAATA |
| 4834-del-R | CGAAGTAGATGATCCGTATGCTGTA |

**Supplementary Table 3. The primer sequences of target genes**

**in mitochondrial functional gene analysis.**

| The name of the primer | primer sequences (5’→3’) |
| --- | --- |
| r-GAPDH-F | AGGTCGGTGTGAACGGATTTG |
| r-GAPDH-R | GGGGTCGTTGATGGCAACA |
| r-ATP5h-F | CAGGCTAGGGTCCGGGAATA |
| r-ATP5h-R | TTCACAGGTTCTCGATGGGC |
| r-Opa1-F | ATTTCGCTCCTGACCTGGAC |
| r-Opa1-R | GGTGTACCCGCAGTGAAGAA |
| r-SDHB-F | CGACCTACAAGGAGAAGCGG |
| r-SDHB-R | TTGAAGGGACTCACGCCAGA |
| r-NDUFB8-F | ACTACGAGCCGTACCCAGAT |
| r-NDUFB8-R | CCCAGTGTATCGGTTCACCC |
| r-UQCRC2-F | AAAGGGCAACTGCTAGAGCC |
| r-UQCRC2-R | TGGGGCAACTTTGAGGGAAT |
| r-COX II-F | GATGACGAGCGACTGTTCCA |
| r-COX II-R | TGGTAACCGCTCAGGTGTTG |
| r-ATP5j-F | TCAGTGCAAGTACCACAGACTC |
| r-ATP5j-R | GAGACTGCTGACCGAAGGAC |
| r-NDUFV2-F | ACCTTGCATGCTTCGAGACT |
| r-NDUFV2-R | CCATCGGTGCGTTTACACAG |
| r-Mfn1-F | TGGTGGCAAACTCGGAATCA |
| r-Mfn1-R  r-ATP5a1-F  r-ATP5a1-R | GAGAGCCGCTCATTCACCTT  TGTTGCTTACCGCCAGATGT  AGCAGGCGAGAGTGTAGGTA |

**
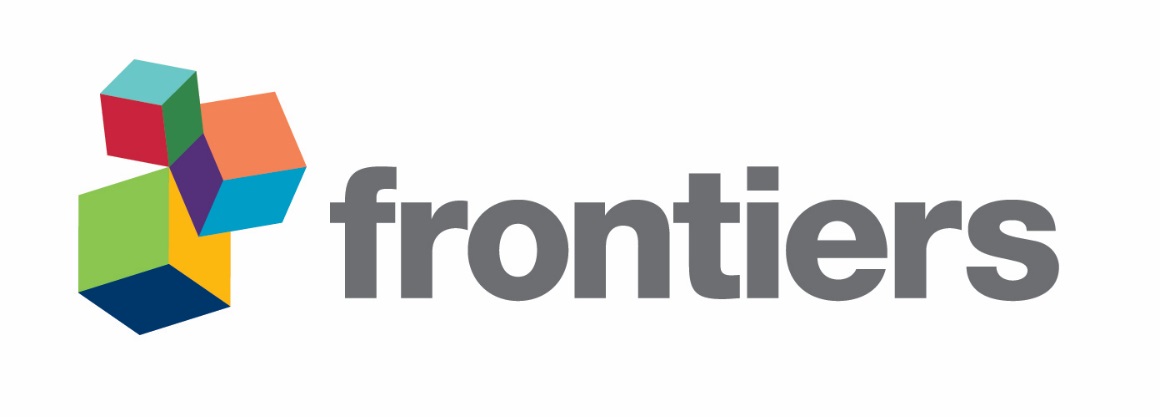
**
